# Exotic catenulid flatworms (Platyhelminthes, Catenulida) and where to find them in a temperate climate – a field study in a botanic garden

**DOI:** 10.3897/zookeys.1296.210918

**Published:** 2026-11-02

**Authors:** Katarzyna Tratkiewicz, Monika Sysiak, Marcin Zych, Ludwik Gąsiorowski

**Affiliations:** 1 Institute of Evolutionary Biology, Faculty of Biology, University of Warsaw, Warsaw, Poland Institute of Evolutionary Biology, Faculty of Biology, University of Warsaw Warsaw Poland; 2 University of Warsaw Botanic Garden, University of Warsaw, Warsaw, Poland University of Warsaw Botanic Garden, University of Warsaw Warsaw Poland

**Keywords:** Artificial habitats, *
Catenula
*, *
Dasyhormus
*, greenhouses, microturbellaria, *
Stenostomum
*, *
Suomina
*

## Abstract

Catenulids are free-living flatworms, common in eutrophic freshwaters such as ponds, ditches, or peatbogs. Although the majority of the species have been described from warmer climates, most molecular studies have been done on animals from temperate zones in Europe. We addressed this gap by sampling for exotic species in greenhouses at the University of Warsaw Botanic Garden and recorded two species known only from tropical areas (*Stenostomum paraguayense* and *Catenula evelinae*) and one exotic species recorded previously from another greenhouse in Poland (*Stenostomum corderoi*). Additionally, in the latter species, evidence is provided for environmentally induced coloration of sensory pits. The collected species were placed on a phylogeny using barcoding of 18S, 28S, and COI genes and paraphyly of the family Catenulidae was retrieved, with *C. evelinae* forming a sister group to the genus *Paracatenula*. In total, six species are recorded, including three with a wide cosmopolitan distribution (*C. turgida*, *S. grande* and *S. tuberculosum*), and provided sequences for five of them, three of which had no previous molecular records (*S. paraguayense*, *C. evelinae* and *S. corderoi*). Greenhouses are confirmed as a useful source of exotic species for taxonomic work on microscopic invertebrates.

## Introduction

Catenulida is an order of free-living microscopic flatworms, occupying a sister position to all of the other platyhelminths (Larsson and Jondelius 2008; Egger et al. 2015; Laumer et al. 2015). Although some families are predominantly marine, most catenulids live in eutrophic freshwaters (Nuttycombe and Waters 1938; Marcus 1945; Larsson and Willems 2010). Most of their diversity to date was reported from tropical habitats, with over half of the species of the largest family, Stenostomidae, noted from South America (Marcus 1945; van der Land 1970; Noreña Janssen 1995; Noreña et al. 2005; Damborenea et al. 2011). Catenulids are found in a diverse range of habitats from lakes and peatbogs (Nuttycombe and Waters 1938; Larsson and Willems 2010) to more specialized environments such as rice fields (Yamazaki et al. 2012) and inside the vase-shaped water-accumulating leaf rosettes in some bromeliads (plant family Bromeliaceae) (Marcus 1945).

Despite their cosmopolitan occurrence in various environments, catenulids remain taxonomically understudied because of difficulties with their identification. Most lack the kind of hard reproductive structures, used extensively in the taxonomy of other flatworms (Brusa et al. 2020). Additionally, they exhibit phenotypic plasticity (Nuttycombe 1956; Rosa et al. 2015; Tratkiewicz et al. 2026), making characters such as shape or size unreliable. Furthermore, particular authors give unequal weight to diagnostic traits, making comparison of species from different geographic regions difficult. All of the above reasons result in a scarcity of comprehensive taxonomical works on catenulids (Nuttycombe and Waters 1938; Marcus 1945; Luther 1960; Kolasa and Young 1974; Noreña et al. 2005), with most of the molecular studies to date performed in temperate climate zones in Europe (Larsson and Jondelius 2008; Diez and Schmidt-Rhaesa 2024; Tratkiewicz et al. 2026). This causes a gap in our understanding of catenulid diversity, which we tried to address in this study by sampling for tropical species at easily accessible localities, i.e., greenhouses in a botanic garden.

Botanic gardens form a very specific environment, often described as ‘tropical islands’ in a temperate climate, where many species are introduced under uncontrolled conditions via components such as soil or plants (Zawierucha et al. 2013; Kolicka et al. 2015). Among the organisms that can thrive in such settings are catenulids, which reproduce asexually through a process called paratomy (Moraczewski 1977; Palmberg 1990; da Rosa and Loreto 2024; Gąsiorowski et al. 2025), allowing even a single individual to establish a new population. In fact, catenulids have been previously researched in greenhouse conditions by Kolasa (1973), who reported several species of *Stenostomum*, *Catenula* and others from a botanic garden in Poznań, Poland (Table 1). One particularly well-studied microhabitat found in greenhouses is the water-filled vase-shaped leaf rosettes of Bromeliaceae plants, which have attracted scientific attention for over a hundred years (e.g., Scott 1912; Richardson 1999; Lopez et al. 2009; Lounibos and Frank 2009; Medeiros et al. 2024). Although some catenulid species have been recorded from bromeliads in natural environments (Marcus 1945), the most recent study of bromeliad fauna in greenhouse conditions by Kolicka et al. (2016) identified no flatworms, rather only several testate amoebae, gastrotrichs, rotifers and other invertebrates in these microreservoirs, a gap we also aimed to address.

**Table 1.** Catenulida representatives previously reported from artificial environments in three localities in Poznań, Poland: Palmhouse, Botanic Garden and Old Zoological Garden.

**Taxon**	**Locality**	**References**
*** Catenula ***		
*C. lemnae* Duges, 1832	Palmhouse: large tank, puddle on a concrete shelf	Kolasa (1973)
*C. macrura* Marcus, 1945	Botanic Garden: *Sphagnum*	Kolasa (1973)
*** Dasyhormus ***		
*D. pygmaeus* (Reisinger, 1924)	Palmhouse: damp soil and Botanic Garden: *Sphagnum*	Kolasa (1973)
*** Myostenostomum ***		
*M. tauricum* (Nasnov, 1924) nomen dubium	Palmhouse: damp soil	Kolasa (1973)
*** Stenostomum ***		
*S. arevaloi* Gieysztor, 1931	Palmhouse: large tank	Kolasa (1973)
*S. constrictum* Papi in Luther 1960	Palmhouse	Kolasa (1973)
*S. corderoi* Marcus, 1945	Palmhouse: mosses	Kolasa and Young (1974)
*S. glandulosum* Kepner & Carter, 1931	Old Zoological Garden: aquarium	Kolasa (1973)
*S. leucops* (Duges, 1828)	Palmhouse	Kolasa (1973)
*S. predatorium* Kepner & Carter, 1931	Palmhouse: puddle on a concrete shelf	Kolasa (1973)
*S. saliens* Kepner & Carter, 1931	Palmhouse: puddle on a concrete shelf, algae from the aquaria walls	Kolasa (1973)
*S. tuberculosum* Nuttycombe & Waters, 1938	Palmhouse: large tank	Kolasa (1973)

In this study, we investigated the occurrence of catenulid flatworms at the University of Warsaw Botanic Garden greenhouses, studying catenulid faunas both in artificial water bodies and associated with bromeliad plants. We provide sequences of three markers (18S, 28S, and COI) for five species, three of which lacked any prior molecular data. We reconstructed their phylogeny and placed them on the Catenulida tree, showing that greenhouses can serve as an important source of microinvertebrate species for taxonomic studies.

## Materials and methods

### Study site

**Figure 1.**
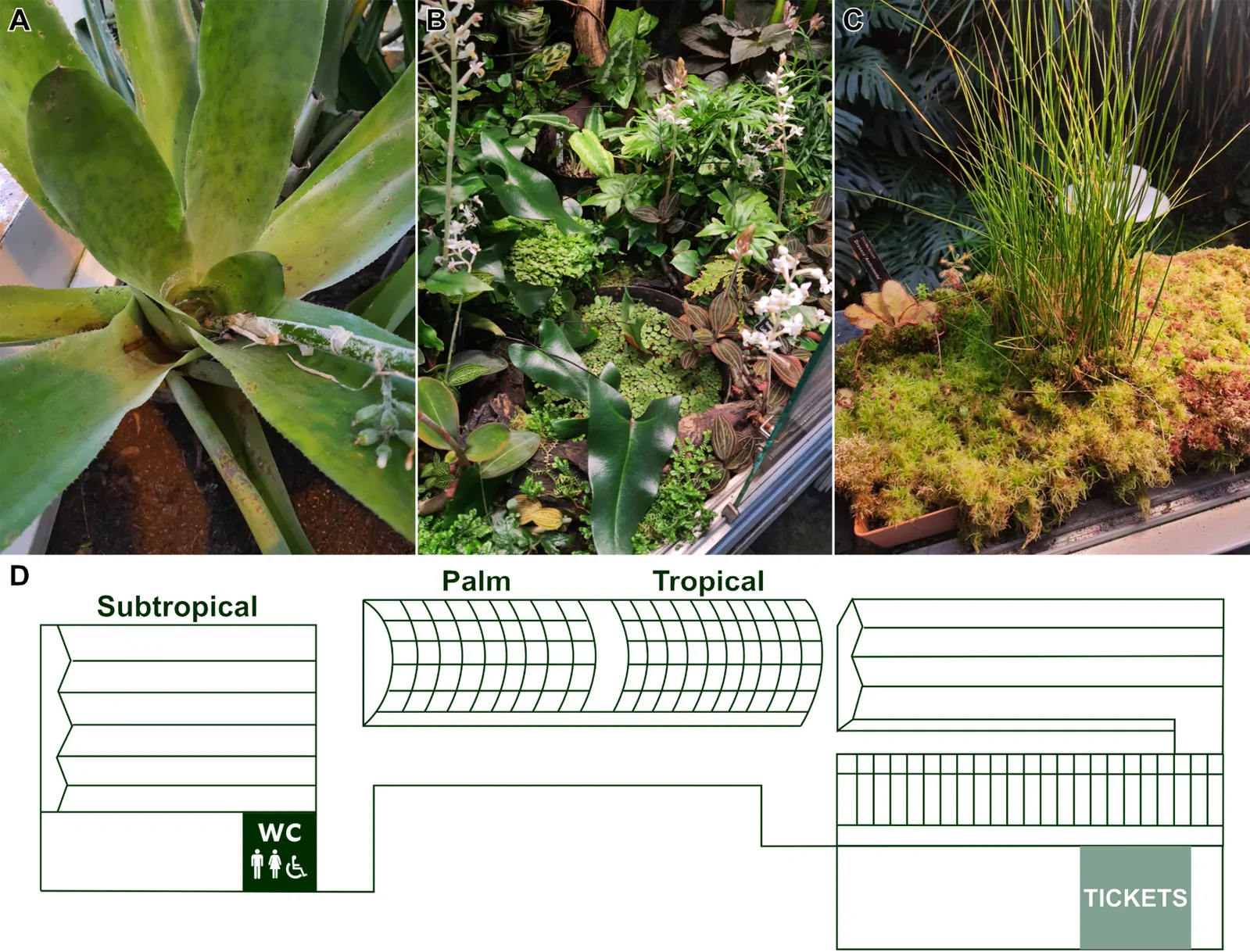
Examples of studied reservoirs and a map of sampling sites. **A**. Inside of *Aechmea lueddemanniana* (Bromeliaceae); **B**. Tank in a cabinet; **C**. *Sphagnum* sp.; **D**. Map of greenhouse buildings.

Sampling was conducted at the University of Warsaw Botanic Garden (Warszawa, Poland). Founded in 1818, it is the third oldest botanic garden in Poland. However, its greenhouses were reconstructed in the late 1940s after being destroyed in 1944. In this study, samples were collected from three different buildings – Subtropical, Palm, and the backside of the Tropical greenhouse (Fig. 1).

### Animal collection and documentation

Sampling was conducted by hand. The water samples were taken into 50 mL Falcon tubes. In the case of Bromeliaceae reservoirs, the water was collected using a plastic Pasteur pipette with a wide tip. For *Sphagnum* extractions, fragments of plants were collected in Ziploc bags, then submerged into distilled water and then squeezed over a 70 μm sieve. During the sampling, an oxygen probe (YSI ProODO YSI) was used to measure the temperature and oxygen concentration at the sample sites (if possible). Afterward, the pH was checked with a pH meter (CP-505 ELMETRON). Details on environmental parameters and species identified in each sampling site are provided in Table 2.

**Table 2.** Catenulid taxa identified in different sampling sites. **A**. Sampling conducted on 23.10.2026 **B**. Sampling conducted on 11.12.2026. The numbers in bold indicate specimen ID.

**Greenhouse**	**Coordinates**	**Sampling site**	**Temp. [°C]**	**pH**	**O_2_ [mg L^-1^]**	**Species**
**A. 23.10.2026 sampling**						
Tropical Greenhouse	52.21663; 21.02753	Display case with vegetation – Tank 1	23.7	7.51	5	*Stenostomum tuberculosum*
		Display case with vegetation – Tank 2	23.4	7.51	1.6	*Stenostomum grande***UW-0048**, *Catenula turgida***UW-0046** and **UW-0056**
		Display case with vegetation – Tank 3	23.3	7.24	6.4	*Stenostomum paraguayense***UW-0050**, *Catenula turgida*
		Display case with vegetation – Tank 4	24.6	7.16	2.2	*Stenostomum corderoi***UW-0062** with red sensory pits
		Inside *Nepenthes* sp.	-	3.88	-	-
		Plastic tank with *Salvinia*	22	6.11	0.9	*Stenostomum corderoi* **UW-0054**
Subtropical Greenhouse	52.21673; 21.02819	Shallow pond (from Łazienki side)	21.6	6.85	3.2	-
		Big pond with fish	27.3	7.6	5.7	-
Palmhouse	52.21666; 21.02785	Pond	21.8	8.31	5.9	-
		Pond	21.8	8.31	5.9	-
		Tank in a display case with vegetation	27.5	8.01	7.2	*Stenostomum grande* **UW-0052**
		Inside leaf rosettes of Bromeliaceae 1	20.8	6.15	4.3	*Stenostomum corderoi* **UW-0057**
		Inside leaf rosettes of Bromeliaceae 2	20.8	6.15	4.3	-
		Inside leaf rosettes of Bromeliaceae 3	20.8	6.15	4.3	-
Outside	52.21672; 21.02741	Pond near Orangery	12.3	6.95	4.9	-
**B. 11.12.2025 sampling**						
Tropical Greenhouse	52.21663; 21.02753	Aquarium with vegetation	24.9	-	8.7	-
		*Sphagnum* sp. 1	-	-	-	*Stenostomum corderoi*
Subtropical Greenhouse	52.21673; 21.02819	*Sphagnum* sp. 2	20 (air)	-	-	*Stenostomum corderoi*, *Catenula evelinae***UW 0068**
		Small aquarium with *Drosera* sp.	20 (air)	-	-	-
Palmhouse	52.21666; 21.02785	Inside leaf rosettes of Bromeliaceae	-	-	-	-

Samples were examined in plastic Petri dishes under the stereomicroscope. Animals were sorted with automatic pipettes and imaged with a Nikon Eclipse NI-SSR microscope equipped with Nomarski contrast optics and a Nikon DS-Ri2 camera. Contrast and brightness of the photographs of the animals were adjusted in the image-analysis software Fiji (Schindelin et al. 2012).

### Animal cultures

One specimen from each location was photographed and sequenced, and if possible, the corresponding morphotype from the same location was used to start an isoline culture (originating asexually from a single individual). Subsequently, individuals from isolines were sequenced to confirm their conspecificity with the originally sequenced morphotype. The isolines are kept alive in the laboratory of Comparative Invertebrate Zoology at the University of Warsaw. The voucher specimens from isoline cultures were fixed in absolute ethanol and deposited in the Museum and Institute of Zoology of the Polish Academy of Sciences in Warsaw: *S. grande* UW-0049 (MIZ PAS PLA000002), *S. corderoi* UW-0061 (MIZ PAS PLA000002), *C. evelinae* (MIZ PAS PLA000003), *C. turgida* (MIZ PAS PLA000004).

### DNA extraction, PCR amplification, and sequencing

After photographic documentation, the DNA was extracted from single worms using Chelex 100 resin (Bio-Rad, Hercules, California, USA) following the procedure from Tratkiewicz et al. (2026). Then, the PCR was performed in a mixture consisting of 2.5 μl of isolated DNA, 0.25 μM of forward and reverse primers, and 12.5 μl of NZT Taq II 23 Green Master Mix (NzyTech, Lisboa, Portugal), in a final volume of 25 μl. The genes were amplified with the following primers: 4fb + 1806R for 18S gene, LSU5 + L1642R for 28S, and COI5B + COI3B for COI (Larsson and Jondelius 2008). The PCR protocol began with 3 minutes of denaturation at 95 °C, followed by 35 cycles comprising 30 s at 94 °C; 30 s at 50 °C (18S), 53 °C (28S) or 43 °C (COI); and 30 s at 72 °C, followed by a final extension at 72 °C for 5 min. The PCR products were purified with Syngen PCR Mini Kit (Syngen Biotech, Wrocław, Poland). Purified DNA was sequenced using the Sanger method by Genomed S.A. (Warsaw, Poland). For the COI gene, the COI5B primer was used for sequencing; the 18S and 28S genes were sequenced with both forward and reverse primers. Sequences were assembled and trimmed using Geneious v. 2026.0.2. Then the sequences were deposited in GenBank under accession numbers PZ143194–PZ143204; PZ164137–PZ164143, and PZ210113–PZ210119.

### Phylogenetic analyses

A dataset combining reference sequences with those obtained in the current study was assembled (Suppl. material 1). The sequences were aligned with MAFFT v. 7.490 (Katoh and Standley 2013) with the automatic (Auto) algorithm setting. Ambiguously aligned regions were trimmed with the Mask Alignment tool in Geneious using a 50% gaps setting. The final concatenated alignment of 3580 nucleotides was divided into 18S, 28S, and COI partitions. The best substitution model for each marker was estimated with ModelTest-NG v. 0.1.7 implemented in RAxML GUI v. 2.0.16 (Edler et al. 2021). The best-fit substitution model selected for 18S and 28S was GTR+I+G, and for COI it was TIM1+I+G. As the TIM1 model is not implemented in MrBayes, the GTR+I+G was applied to all three partitions. The Bayesian phylogeny was reconstructed using MrBayes v. 3.2.7 (Ronquist et al. 2012) employing default priors and the same partitioning scheme. Two simultaneous analyses were conducted, each comprising 2 million generations with one cold and three heated chains, sampling every 1000 generations. Tracer v. 1.5 (Rambaut et al. 2018) was utilized to assess the convergence of the Markov Chain Monte Carlo (MCMC) chains.

## Results

### Morphology of sampled species

Our collection yielded nine barcoded specimens, representing five morphotypes, belonging to two genera: two species of *Catenula* and three species of *Stenostomum*. One *Stenostomum* species was photographed but not barcoded.

#### Catenula turgida

Taxon classificationAnimaliaPlatyhelminthesCatenulidae

(Zacharias, 1902)

0B19946C-1BD9-5B42-9B27-2CAC0D3B1CBB

Fig. 2A–D

##### Sampling sites.

Display case with vegetation – Tanks 2 and 3.

**Figure 2.**
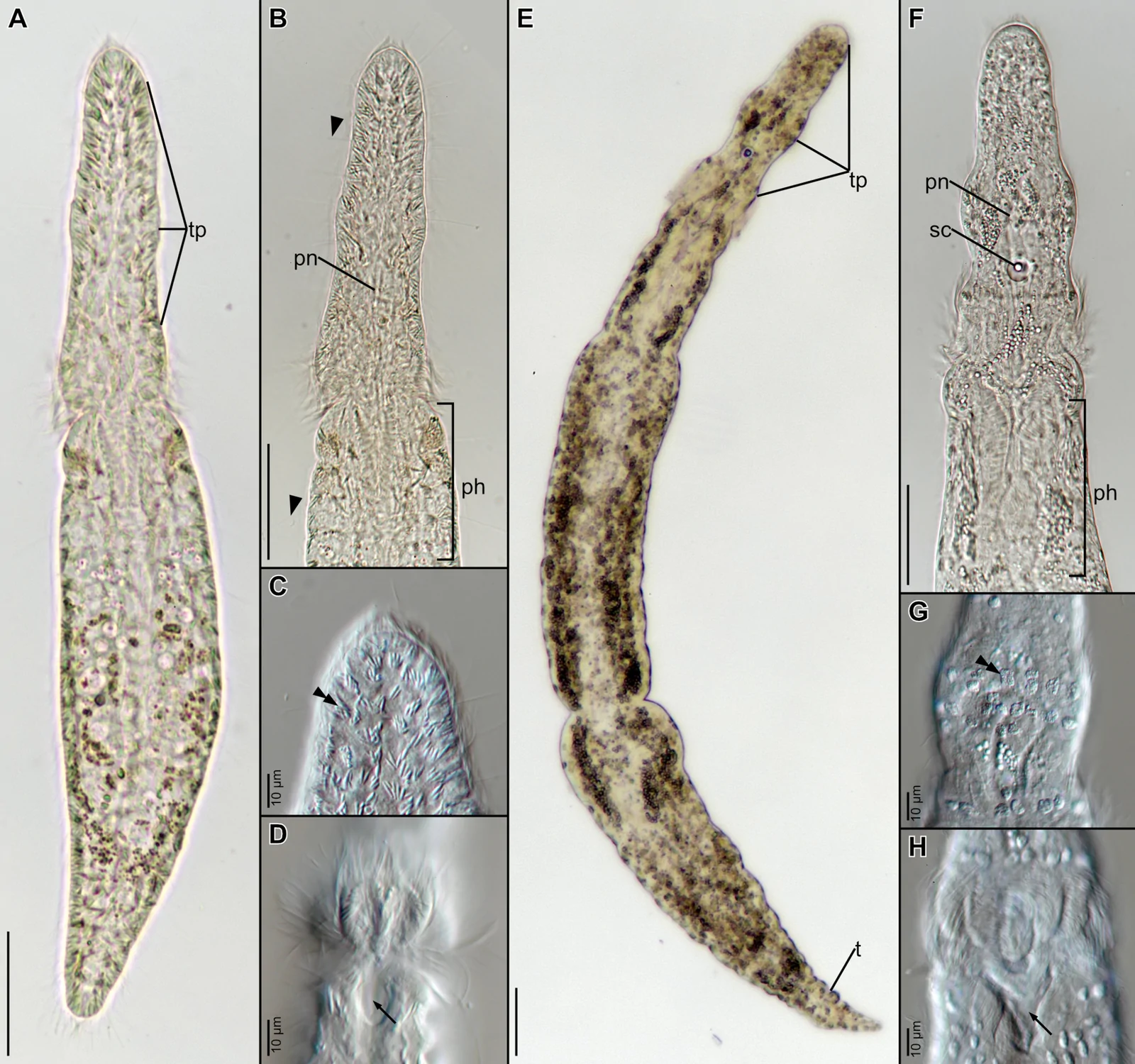
Morphology of a *Catenula turgida* (**A–D**) and *C. evelinae* (**E–H**). **A, E**. Overview of the animal; **B, F**. Overview of the head; **C, G**. Magnification of the rhabdoids; **D, H**. Magnification of the ciliated ring in the mouth region. Abbreviations: ph, pharynx; pn, protonephridial canal; sc, statocyst; t, tail; tp, tripartition of the head; arrow, mouth opening; arrowhead, long stiff cilia; double arrowhead, rhabdoids/epidermal inclusions. Scale bars: 50 μm (**A, B, E, F**); 10 μm (**C, D, G, H**).

##### Description.

A *Catenula* reaching 400 μm in length. The body shape is conical, with a narrowing posterior part (Fig. 2A). The head is clearly separated from the rest of the body, with a slight tripartition (Fig. 2A). In our specimens, we could not observe a statocyst. At the base of the head, a ring of cilia is present (Fig. 2D) with four furrows on the ventral side. The ciliated ring is followed by a small triangular mouth opening and a long pharynx with cilia arranged like a comb (Fig. 2B). The gut does not reach the posterior end of the body, which is blunt, without a tail. The protonephridial canal is visible throughout the whole length of an animal and ends in the caudal region. In the epidermis, around 3 μm long rod-shaped rhabdoids occur, grouped into sets of a few (Fig. 2C). The animal is covered by a uniform layer of cilia, with longer, stiffer ones distributed sparsely but evenly around the body (Fig. 2B).

The worms can be cultured with some difficulty on Chalkley’s Medium with wheat grain at 20 °C. In the cultures, they swim freely in the water column or crawl in the sediment at the bottom of the dish and prey on unidentified bacteria.

##### Distribution.

Germany, *Sphagnum* in moorland near Plön (Zacharias 1902); Brazil, bromeliads and stream in São Paulo (Marcus 1945); Finland, raised bog in Tvärminne (Luther 1960); Sweden, *Sphagnum* from various locations (Larsson and Willems 2010); Brazil, wetland in Rio Grande do Sul (Braccini et al. 2017) – however, the general body shape of the last is more reminiscent of *C. sawayai*.

#### Catenula evelinae

Taxon classificationAnimaliaPlatyhelminthesCatenulidae

Marcus, 1945

32BF3CA4-4127-5AA4-A8DC-DCCF780E270C

Fig. 2E–H

##### Sampling site.

*Sphagnum* sp. 2.

##### Description.

Animals reach a length of 800 μm in the two-zooid stage; chains longer than two zooids were not observed. The body is cylindrical, with a narrowing and pointed posterior end (Fig. 2E). The head is not separated from the body; however, it has a clearly distinguishable tripartition (Fig. 2E). The first thickening at the head occupies the anterior tip, and the last one lies in front of the mouth. On each of the three parts, rounded granular epidermal inclusions are present (Fig. 2G). The third thickening is surrounded by a ciliated ring (Fig. 2H) split into four furrows on the ventral side. The mouth opening is located behind the ciliated ring, leading to a pharynx shaped like a comb, similar to the one in *C. turgida*. Between the second and third thickening, there is a statocyst with a single, spherical statolith (Fig. 2F). The gut was brownish in our specimens, but the inner lining of the intestine was not clearly distinguishable. The posterior end of the body is crenulated and resembles a tail (Fig. 2E). The cilia layer is uniform and covers the whole animal; no stiff cilia were observed.

The worms are very easily culturable on Chalkley’s Medium with wheat grain at 20 °C. In the cultures, they swim freely in the water column or crawl in the sediment at the bottom of the dish and prey on bacteria and small protozoans.

##### Distribution.

Brazil, bromeliads from São Paulo, Pinheiros River meadow (Marcus 1945); Brazil, wetland in Rio Grande do Sul (Braccini et al. 2017).

#### Stenostomum corderoi

Taxon classificationAnimaliaPlatyhelminthesStenostomidae

Marcus, 1945

1734085A-4AFC-5FFF-8BF3-DF16FD46F215

Fig. 3A–H

##### Sampling sites.

Display case with vegetation – Tank 4 (red pits), Plastic tank with *Salvinia* sp., Inside leaf rosettes of Bromeliaceae 1, *Sphagnum* sp. 1, *Sphagnum* sp. 2.

##### Description.

**Figure 3.**
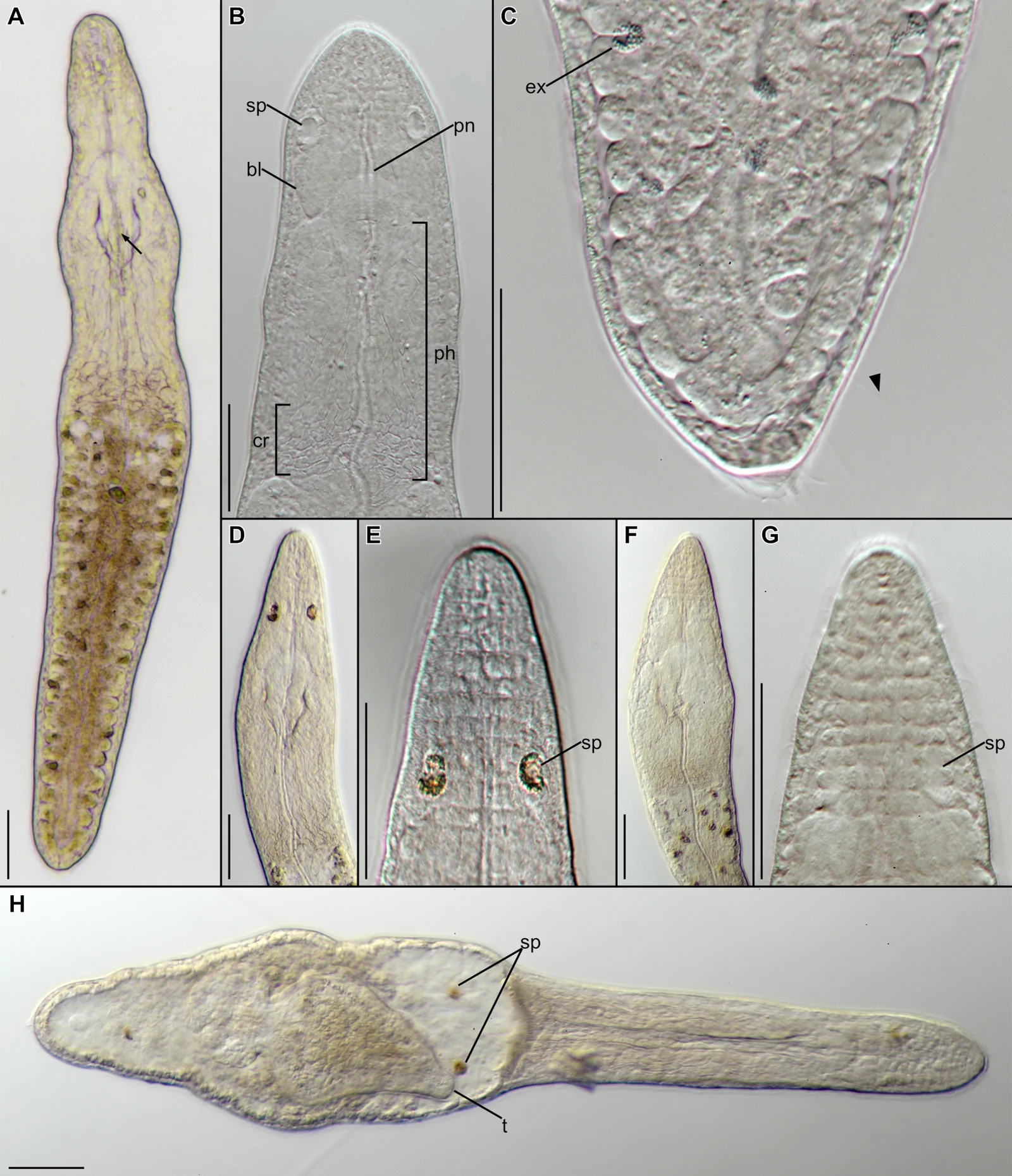
Morphology of a *Stenostomum corderoi* from Tank with *Salvinia* (**A–C**) and the individuals with red sensory pits from Cabinet – Tank 4 (**D–H**). **A**. Overview of an entire worm; **B**. Magnified view of the head; **C**. Magnified view of the caudal region; **D**. Overview of the head of the individual with red sensory pits from Cabinet – Tank 4; **E**. Magnification of the sensory pits of the same individual; **F**. Overview of the head of the morphotype from Cabinet – Tank 4 put in water from Tank with *Salvinia* for 24 h; **G**. Magnification of the sensory pits of the same individual; **H**. Cannibalism in *S. corderoi*. Sensory pits and tail of the predated conspecific worm are visible inside the gut. Abbreviations: bl, brain lobe; cr, creased area of the pharynx; sp, sensory pit; t, tail; ex, excretophores; ph, pharynx; pn, protonephridial canal; arrow, mouth opening; arrowhead, long stiff cilia. All scale bars equal 50 μm.

Single worms reach up to 600 μm in length (Fig. 3A), often occurring in longer chains consisting of up to five zooids. The body is cylindrical, narrowed in the anterior part and rounded at the posterior end with a noticeably wider pharyngeal region. The sensory pits are small and round, enclosed in the head, located in front of the brain lobes (Fig. 3B). The animals lack refracting bodies. The pits and brain arrangement are similar to those of *S. brevipharyngium* and *S. saliens*, but the species is distinguishable by its pharyngeal region. The mouth opening is V-shaped, extending along half of the pharynx where it is glandular, with the rest of the pharynx having a characteristic creased surface (Fig. 3B). Excretophores appear in the gut, which has a characteristic wavy margin (Fig. 3C). The ciliary coat is uniform, with a few stiffer cilia distributed across the body. At the very posterior part, a tuft of normal cilia appears (Fig. 3C).

Some individuals found in Cabinet – Tank 4 exhibited red sensory pits, noticeable even under the dissecting microscope (Fig. 3D, E). Initially thought to represent a different species, these individuals were, upon closer examination, found to exhibit morphology consistent with *S. corderoi*. Animals were separated and moved into filtered water from another location: a tank with *Salvinia*. After 24 h in the water from the *Salvinia* tank, the coloring of the pits disappeared (Fig. 3F, G). Later, two animals from each location – both Tank 4 and the *Salvinia* tank were separated and put in the 4-well plate. One of the animals was kept in the original filtered water, and the other one was put in the filtered water from the other location. After one day in changed water, the animal with red pits (Tank 4) lost the coloration, and the animal that exhibited normal pits (Tank with *Salvinia*) gained the red color. The color of the pits in control animals in filtered water from the same location did not change.

The worms are easily culturable on Chalkley’s Medium with wheat grain at 20 °C. They swim freely in the water column and prey on small protozoans. When starved, a few instances of cannibalism were observed (Fig. 3H).

##### Distribution.

Brazil, bromeliads from São Paulo (Marcus 1945); France (De Beauchamp 1948); Poland, mosses from Poznań Palmhouse (Kolasa and Young 1974).

#### Stenostomum tuberculosum

Taxon classificationAnimaliaPlatyhelminthesStenostomidae

Nuttycombe & Waters, 1938

64F68772-0DE6-5B97-975C-D9372C687E03

Fig. 4A, B

##### Sampling site.

Display case with vegetation – Tank 1.

##### Description.

**Figure 4.**
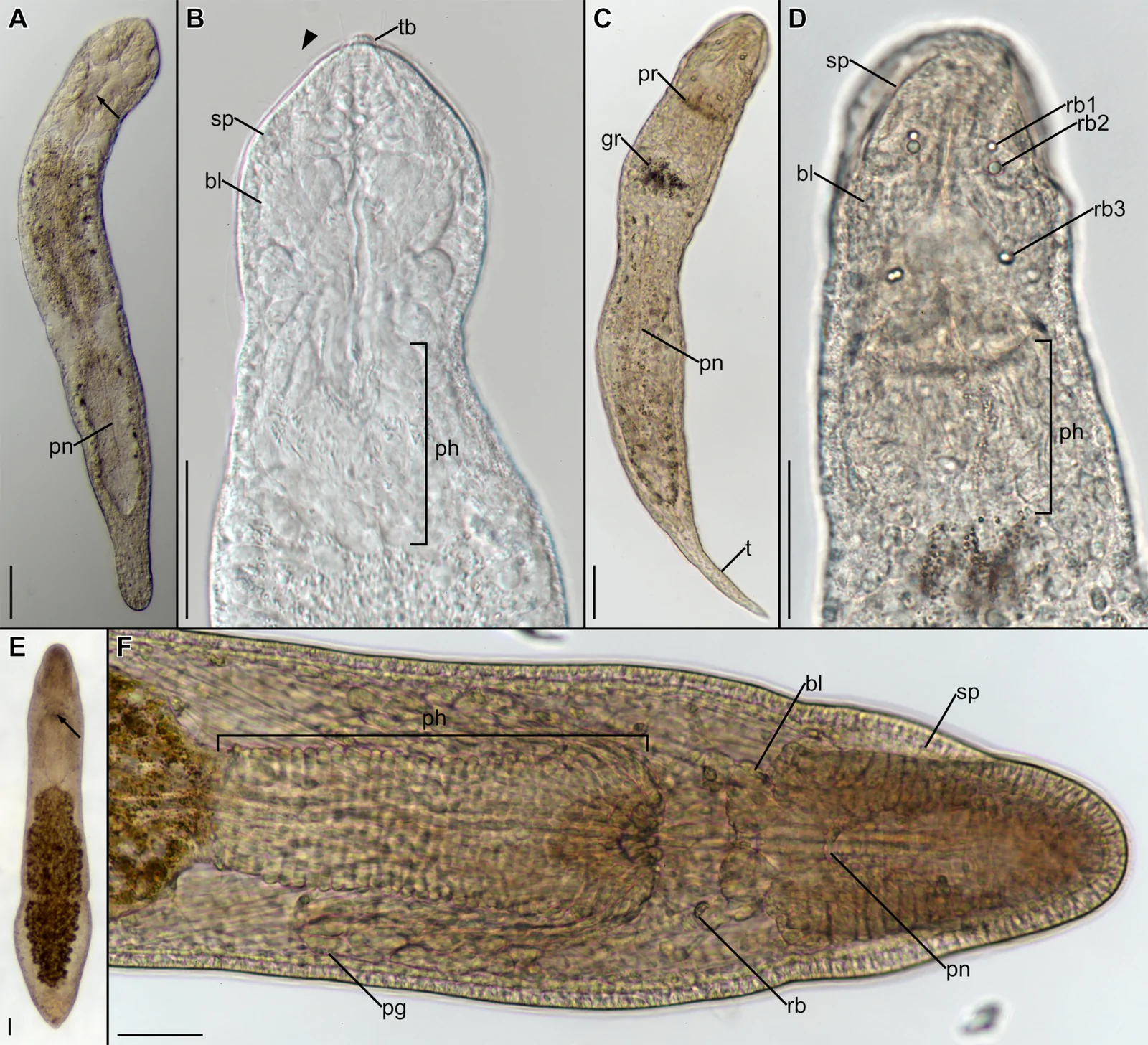
Morphology of a *Stenostomum tuberculosum* (**A, B**), *S. paraguayense* (**C, D**) and *S. grande* (**E, F**). **A**. Overview of *S. tuberculosum*; **B**. Magnification of the head of *S. tuberculosum*; **C**. Overview of *S. paraguayense*; **D**. Magnification of the head of *S. paraguayense*; **E**. Overview of *S. grande*; **F**. Magnification of the head of *S. grande*. Abbreviations: bl, brain lobe; sp, sensory pit; gr, gut ring; pg, pharyngeal gland; ph, pharynx; pn, protonephridium; pr, pharyngeal ring; rb, refracting body; t, tail; tb, tuberculum; arrow, mouth opening; arrowhead, long stiff cilia. All scale bars equal 50 μm.

A *Stenostomum*, reaching a length of 600 μm in two-zooid individuals (Fig. 4A). The head is triangular with a characteristic tubercle at the very top (Fig. 4B). Sensory pits are directed anteriorly, deeply enclosed in the head. The pits are connected to the posterior brain lobes, which are oval (Fig. 4B). The animals lack refracting bodies. The mouth opening is V-shaped and strongly muscular (Fig. 4A). The pharynx is relatively short with fine granular gland cells, the refracting elements described by Marcus (1945) (Fig. 4B). The posterior end of the gut does not extend to the caudal end, which is narrower than the rest of the body. The protonephridium is straight, with a terminal opening at the very end of the animal.

The worms survived for only a few days in Chalkley’s Medium with wheat grain before the culture collapsed. We did not succeed in further attempts at culturing them.

##### Distribution.

US, Mountain Lake, Virginia, and pond at Athens, Georgia (Nuttycombe and Waters 1938); Brazil, streams in São Paulo (Marcus 1945); Poland, tanks in Poznań Palmhouse (Kolasa 1973); Surinam, Suriname River near Paramaribo (van der Land 1970); Germany and Finland (Lanfranchi and Papi 1978); Argentina, Buenos Aires (Noreña et al. 2005); Japan, Ikeura rice field (Yamazaki et al. 2012); Brazil, ESEC Taim water channel (Reyes et al. 2021).

#### Stenostomum paraguayense

Taxon classificationAnimaliaPlatyhelminthesStenostomidae

(Martin, 1938)

AB71284D-1569-5B22-96F3-788F10428302

Fig. 4C, D

##### Sampling site.

Display case with vegetation – Tank 3.

##### Description.

A *Stenostomum* reaching the length of 500 μm in a single zooid stage. The body is cylindrical with a distinguishable tail and a rounded anterior part (Fig. 4C). The sensory pits are elongated and connected to the anterior, metameric brain lobes (Fig. 4D). There are three pairs of refracting bodies. The first pair occurs at the middle of the length of the pits, the second at the end of the pits, and both consist of a single sphere. The third pair is connected to the end of the posterior brain lobes and is made of two connected spheres (Fig. 4D). The mouth opening is small and slender, not easily recognizable on the photos; however, it is accompanied by a very distinguishable ring of darker cells (Fig. 4C). The short pharynx passes into another ring of darker cells at the beginning of the intestine. The presence of these two dark rings is characteristic of the species. The body is yellowish with a uniform layer of cilia. The gut does not reach the caudal end of the body, which ends in a long tail (Fig. 4C).

This species is highly similar to *S. arevaloi*; however, according to Marcus (1945), it differs in width, mouth shape, gland cells at the beginning of the intestine, and dorsocaudal appendix. We did not observe any easily distinguishable appendix, but according to Marcus, the structure is not always present.

The animal can survive for a few days in Chalkley’s Medium, fed on rotifers *Lecane inermis*, but the worms progressively decreased in size and died, likely due to starvation. We did not succeed in attempts of culturing them.

##### Distribution.

Brazil, São Paulo tanks and aquariums, Pinheiros River meadow, Canindé lake (Marcus 1945); Argentina, Córdoba (Adami and Damborenea 2021) – described as *S. arevaloi* but very likely *S. paraguayense*.

#### Stenostomum grande

Taxon classificationAnimaliaPlatyhelminthesStenostomidae

Child, 1902

E58B6F51-4AB0-5A6E-BA29-0F8C94432535

Fig. 4E, F

##### Sampling sites.

Display case with vegetation – Tank 2, Display case with vegetation in a Palmhouse.

##### Description.

A large *Stenostomum* reaching the length of 1100 μm in a two-zooid individual. The body shape is cylindrical and compact with pointed anterior and posterior parts (Fig. 4E). The sensory pits are elongated and open to the environment, similar to the ones in *S. paraguayense*. They are enclosed shallowly in the anterior brain, which is composed of many metamers (Fig. 4F). The posterior brain is made from four lobes with the posterior ones connecting through protrusions with refracting bodies, which are tear-shaped and consist of many granules (Fig. 4F). The mouth opening is small but clearly visible and followed by a long pharynx, with strong circular musculature and a large number of glandular cells along the anterior two-thirds of its length (Fig. 4F). The gut is dark brown and does not reach the caudal end of the body, which ends in a small, triangular tail (Fig. 4E). The protonephridium is clearly visible, even at the dissecting scope, and ends after the gut, but does not reach the posterior extremity of the body.

The worms can be cultured on Chalkley’s Medium with wheat at 20 °C, where the animals stay at the bottom of a dish, feeding on decomposing organic matter.

##### Distribution.

US, New York and Massachusetts (Graff 1913); Russia, Kola Peninsula (Nassonov 1924); US, Virginia (Nuttycombe and Waters 1938); Brazil, São Paulo (Marcus 1945); Surinam, Onverwacht (van der Land 1970); Poland, heated Konin Lakes (Kolasa 1977); US, New York (Kolasa et al. 1987); Japan, Ikeura rice field (Yamazaki et al. 2012); Argentina, Buenos Aires (Noreña et al. 2005); Lake Mangueira and Lake Nicola (Reyes et al. 2021).

### Phylogenetic position of sampled species

**Figure 5.**
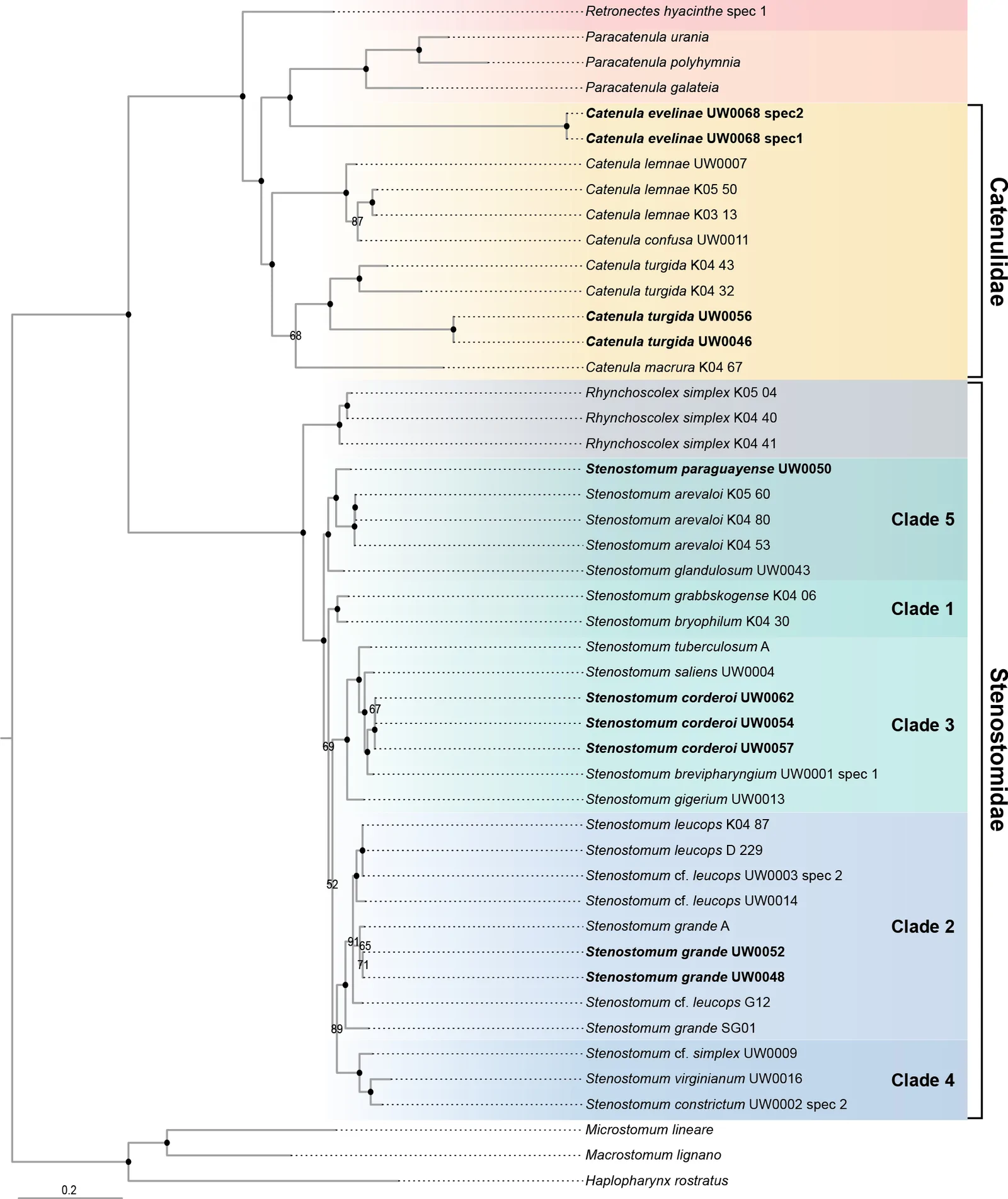
Consensus tree of Catenulida inferred with a Bayesian approach from the concatenated 18S, 28S and COI datasets. Filled dots indicate posterior probability > 95%, lower values are provided. Terminals in bold indicate newly sequenced individuals. Different genera are colored: *Retronectes* in red, *Paracatenula* in orange, *Catenula* in yellow, *Rhynchoscolex* in grey and *Stenostomum* in greens or blues. Different clades of *Stenostomum* following Tratkiewicz et al. (2026) and a newly defined Clade 5 are marked.

Bayesian analysis recovered a monophyletic Catenulida, divided into two clades (Fig. 5). The first clade consisted of the family Stenostomidae (*Stenostomum* and *Rhynchoscolex*), and the second of Catenulidae, Paracatenulidae and Retronectidae. The family Catenulidae was recovered as paraphyletic, as specimens identified morphologically as *C. evelinae* (Catenulidae) grouped with high support with *Paracatenula* (Paracatenulidae), and not with the members of the genus *Catenula* (Catenulidae). Specimens of *C. turgida* grouped with the conspecifics worms from Sweden. *Stenostomum* clade shows a topology congruent with that of Tratkiewicz et al. (2026), with four well-supported clades (Clades 1–4) and poorly resolved deep nodes. *Stenostomum paraguayense* grouped closely with *S. arevaloi* Gieysztor, 1931 and *S. glandulosum* Kepner & Carter, 1931, forming a sister group to all the remaining *Stenostomum* (here after referred to as Stenostomidae Clade 5). *S. corderoi* was placed within Stenostomidae Clade 4 (with *S. brevipharyngium* Kepner & Carter, 1931 as its closest relative) and *S. grande* in the Stenostomidae Clade 2 (grouping with *S. grande* from Japan).

## Discussion

### Catenulid diversity in greenhouses

As reported in this study, greenhouses create suitable conditions for a variety of catenulid species. Although the routes by which they colonize botanic gardens remain unknown, tropical species may be transported with wet soil or plants. Borkott (1970) observed egg-laying in catenulids, but it is not known whether these eggs serve as dormant stages capable of withstanding extreme or unfavorable conditions, for example during long-distance transport. In total, we recorded six different catenulid species from the University of Warsaw Botanic Garden: three widely distributed around the world (*C. turgida*, *S. tuberculosum*, *S. grande*), one exotic species recorded previously from another greenhouse in Poland by Kolasa and Young (1974) (*S. corderoi*), and two known so far exclusively from tropical areas (*C. evelinae*, *S. paraguayense*). For the latter three, we provided the first reference molecular sequences for several barcodes. We report a similar species composition to that previously found in studies of botanic gardens. Besides *S. corderoi*, we recorded *S. tuberculosum*, which has been previously found in a Poznań Palmhouse, Poland (Kolasa 1973). Although we did not succeed in sequencing this species, its characteristic morphological feature (the tubercle) allowed us to identify it with a high degree of confidence. Another species recorded by Kolasa was *Dasyhormus pygmaeus*, which we argue is similar to *C. evelinae*; this is discussed further in the final part of the Discussion.

We did not observe any clear pattern regarding the environmental preferences of catenulids. They were found across a diverse range of pH from 6.11 to 8.01 and oxygen levels from 0.9 to 7.2 mg/l. No catenulids were found in the sample with the lowest pH recorded (3.88 in *Nepenthes*), suggesting they are not adapted to extremely low pH. *Stenostomum corderoi* was found in the tank with the lowest oxygen concentration in the study (0.9 mg/l), suggesting this species may be tolerant of microxic conditions.

### Catenulids and bromeliads

The association between bromeliads and invertebrates has a long evolutionary history, estimated to have formed around 16 million years ago (Balke et al. 2008). Consequently, both the invertebrate and vertebrate fauna of bromeliads have been the subject of numerous studies (e.g., Scott 1912; Richardson 1999; Lopez et al. 2009; Lounibos and Frank 2009; Medeiros et al. 2024), including a study of artificial bromeliad environments conducted in Poland by Kolicka et al. (2016), where turbellarian representatives were found but not identified to species or genus level. Some animal species are known to inhabit bromeliads exclusively, for instance the acari *Steneotarsonemus ananas* (Tryon, 1898) *sensu* Beer, 1954 or the gastrotrich Chaetonotus (Hystricochaetonotus) furcatus Kisielewski, 1991, but many species found in bromeliads are just opportunistic visitors. Although no catenulid species is known exclusively from bromeliads, we included these habitats in our study because some species have previously been recorded from them in the wild. Notably, Marcus (1945) reported in his revision of Brazilian microturbellarians, four catenulid species from bromeliads: *Catenula evelinae*, *Stenostomum anatirostrum*, *Stenostomum virginianum*, and *Stenostomum corderoi*. While the first three were also found in other habitats, the last was reported exclusively from bromeliads. In our study, *S. corderoi* was likewise found in *Bromelia*, but it also occurred in small tanks and *Sphagnum* samples, making it the most common species across all sampling sites (Table 2). Although bromeliads were the only habitat reported in the original description of this species, it was later also recorded from mosses in a botanic garden (Kolasa and Young 1974). Given that bromeliads remain the only known natural habitat for *S. corderoi*, we speculate that the species is closely associated with them and may have been transported to greenhouses via Bromeliaceae plants, later colonizing other available habitats. Definitive conclusions, however, would require further sampling in its native range.

### Phenotypic plasticity in catenulids

Catenulids exhibit exceptional phenotypic plasticity, making traits such as body size or shape unreliable for taxonomy (Nuttycombe 1956; Rosa et al. 2015; Tratkiewicz et al. 2026). During this study, we found a small population of *S. corderoi* with red sensory pits in one of the tanks. Aside from the brightly pigmented pits, these animals were morphologically indistinguishable from specimens of *S. corderoi* found in the same cabinet but in different tanks. Subsequent sequencing confirmed they belonged to the same species, a finding further supported by the loss of pit coloration after the animals were transferred to water from a different tank. The pits are filled with a mucus-like substance (Ott 1892; Kepner and Cash 1915; Reuter et al. 1993; Gąsiorowski et al. 2023), and we therefore hypothesize that they can accumulate particulate matter or pigmented compounds dissolved or suspended in the water, causing the observed change in coloration. The genus *Xenostenostomum*, described by Reisinger (1976), was characterized by red, elongated pits, which could also have resulted from pigment accumulation, although this remains speculative as no photographs or drawings of this taxon exist. In light of our findings, we propose that pit coloration should not be used as a taxonomically relevant character in catenulids.

### New insights into catenulid phylogeny

In our previous study, *Stenostomum* was split into four clades (Tratkiewicz et al. 2026), for which distinct morphological traits can be identified that reflect phylogenetic position. The newly sequenced species obtained in the course of this study aligned with the previously characterized clades. For instance, *S. corderoi* exhibits all the characteristics of Clade 3: the absence of refracting bodies, a V-shaped mouth with lips, and closed sensory pits. Furthermore, *S. arevaloi* (previously an isolated lineage) now groups with *S. paraguayense* and *S. glandulosum* in Clade 5. This clade also shares common features, such as multiple pairs of refracting bodies and open, slit-like sensory pits (the presence of these traits in *S. glandulosum* is documented by Kepner (1930), and in *S. arevaloi* by (Marcus 1945)). Consequently, the systematics of the genus *Stenostomum* have largely stabilised, with five distinguishable clades supported by both molecular and morphological data. The newly sequenced species fit neatly into these clades, aligning with their respective morphological characteristics.

Another notable result of our study concerns the phylogenetic relationships within the family Catenulidae. We recorded animals which we identified as *Catenula evelinae*. Due to the presence of a statocyst, a ciliated ring around the mouth, and characteristic round epidermal inclusions, we are confident in our identification of these specimens as *C. evelinae* sensu Marcus (1945). This species was first described by Marcus (1945) and placed in the genus *Suomina* alongside *S. turgida* and *S. sawayai*. Later, Larsson and Jondelius (2008) moved *Suomina* into *Catenula*, based on the molecular data from *C. turgida*, which was recovered in a monophyletic position within the *Catenula* clade. In the present study, *C. turgida* was retrieved in a similar position within *Catenula*; however, *C. evelinae* grouped closer to *Paracatenula*. *Catenula* species are more rapidly evolving than *Stenostomum*, which is reflected in the longer branches. Combined with the poor sampling of this part of the tree, this can make the phylogenetic reconstruction prone to errors. Elucidating the exact position of *C. evelinae* will require denser sampling of the other genera on this branch of the catenulid tree – particularly sequencing of genera currently lacking any molecular data, such as *Dasyhormus*, *Chordarium* and *Africatenula*, as well as additional species of *Catenula*, especially *C. sawayai* (originally described as *Suomina*). Based on the data we provided, it appears that *C. evelinae* should be placed in a separate genus from *Catenula*. However, as we lack sufficient molecular data, we are withholding a final taxonomic decision until the tree is supplemented with additional species and further supported by multi-gene analyses.

We also speculate on the similarity between our specimens of *C. evelinae* and *Dasyhormus pygmaeus* recorded by Kolasa (1973). The original description of this species was provided by Reisinger (1924) as *Catenula pygmaeus*. Although the description is vague and lacks drawings, Reisinger reports characteristics that we also observed in our specimens, including a statocyst, a ring of cilia, a wrinkled tail, and asexual reproduction with a maximum of two zooids. Marcus (1945) later described *Dasyhormus lithophorus* and noted its similarity to *C. pygmaeus*, though the absence of drawings and the different habitat of those specimens make the comparison uncertain. The third and final description of what is now *Dasyhormus pygmaeus* was provided by Kolasa (1973), in his botanic garden studies. The similarity between our specimens and Kolasa’s drawing, combined with the comparable habitat (*Sphagnum* samples from an exotic greenhouse in both cases), leads us to question the correct identification of this *Dasyhormus* record. Given the vague original description of *D. pygmaeus* and its sparse records – particularly the lack of reliable drawings or photographic documentation, we consider this species a nomen dubium. Nevertheless, recording and sequencing of *D. lithophorus* as described by Marcus could help determine its relationships within Catenulida and clarify the exact phylogenetic relationship between *C. evelinae* and the genus *Dasyhormus*.

## Conclusions

Greenhouses, regarded as tropical islands in temperate zones, can serve as a source of non-native animal species characteristic of warmer habitats, and our results confirm that they represent a source of exotic species for barcoding and taxonomic studies. In particular, we provided new data for two species recorded previously only from tropical areas – *S. paraguayense* and *C. evelinae*. One limitation of this type of research is establishing the origin of species, which can be especially difficult for taxa with limited biogeographic data, such as microturbellaria. Nevertheless, specimens obtained from botanic gardens can provide valuable material for taxonomic work on microturbellaria, as demonstrated here. Overall, our data suggest that in temperate climates, sampling in greenhouses is a simple and cost-effective complement to traditional field studies, particularly for barcoding of microscopic invertebrates.

## Supplementary Material

XML Treatment for Catenula turgida

XML Treatment for Catenula evelinae

XML Treatment for Stenostomum corderoi

XML Treatment for Stenostomum tuberculosum

XML Treatment for Stenostomum paraguayense

XML Treatment for Stenostomum grande

## Additional information

### Artificial Intelligence (AI) use

The authors accept full responsibility for the content of the manuscript, including the disclosure of any use of AI.

No AI tools were used in the preparation of this manuscript.

### Author ORCIDs

K. Tratkiewicz https://orcid.org/0009-0007-1990-0307

M. Sysiak https://orcid.org/0009-0009-2990-9764

M. Zych https://orcid.org/0000-0001-6961-069X

L. Gąsiorowski https://orcid.org/0000-0003-2238-7587

### Data availability

All of the data that support the findings of this study are available in the main text or Supplementary Information. The molecular data underlying this article are available in GenBank under accession numbers PZ143194–PZ143204; PZ164137–PZ164143 and PZ210113–PZ210119. The voucher specimens were deposited in Museum and Institute of Zoology of the Polish Academy of Sciences in Warsaw with ID numbers MIZ PAS PLA000001-MIZ PAS PLA000004.

## Supplementary materials

Supplementary material 1 Set of reference sequences used in phylogenetic analyses Data type xlsx https://binary.pensoft.net/file/1826874This dataset is made available under the Open Database License (http://opendatacommons.org/licenses/odbl/1.0/). The Open Database License (ODbL) is a license agreement intended to allow users to freely share, modify, and use this Dataset while maintaining this same freedom for others, provided that the original source and author(s) are credited. Katarzyna Tratkiewicz, Monika Sysiak, Marcin Zych, Ludwik Gąsiorowski
